# Prognostic significance of residual nodal disease after neoadjuvant endocrine therapy for hormone receptor-positive breast cancer

**DOI:** 10.1038/s41523-020-00177-6

**Published:** 2020-08-13

**Authors:** Olga Kantor, Stephanie Wong, Anna Weiss, Otto Metzger, Elizabeth A. Mittendorf, Tari A. King

**Affiliations:** 1 Division of Breast Surgery, Department of Surgery, Brigham and Women’s Hospital, Boston, MA USA; 2 Breast Oncology Program, Dana-Farber/Brigham and Women’s Cancer Center, Boston, MA USA; 3 McGill University Health Centre, Montreal, QC Canada; 4 Medical Oncology, Dana-Farber Cancer Institute, Boston, MA USA

**Keywords:** Breast cancer, Breast cancer

## Abstract

Axillary management after NET has not been well studied and the significance of residual axillary node disease after NET remains uncertain. We used the National Cancer Data Base to examine the prognostic significance of residual nodal disease after NET. From 2010–2016, 4,496 patients received NET for cT1–3N0–1M0 hormone receptor-positive, HER2-negative breast cancer. Among cN0 patients treated with NET, final node status was ypN0 in 65%, isolated tumor cells (ITCs) in 3%, ypN1mi in 6%, and ypN1 in 26%. In cN1 patients, nodal pathologic complete response was uncommon (10%), and residual nodal disease included ITCs in 1%, ypN1mi in 3%, and ypN1 in 86%. There were no differences in 5-year overall survival (OS) between patients with pathologic node-negative disease, ITCs, or micrometastases after NET. When compared to a matched cohort of upfront surgery patients, there were also no differences in 5-year OS between NET and upfront surgery patients for any residual nodal disease category. These findings suggest NET patient outcomes mirror those of upfront surgery patients and present an opportunity to consider de-escalation of axillary management strategies in NET patients.

## Introduction

In the era of biologic markers and genomic assays, the use of neoadjuvant endocrine therapy (NET) for select patients with hormone receptor-positive, HER2-negative (HR+HER2−) breast cancer is gaining popularity^1^. Although pathologic complete response (pCR) is rare with NET, clinical response rates do result in increased eligibility for breast conserving therapy (BCT)^2–6^ and randomized trials comparing NET to neoadjuvant chemotherapy (NAC) have shown similar rates of (BCT with decreased toxicity for patients randomized to NET^7–10^. Coupled with results of recent trials using genomic assays to define broader populations that will not benefit from the addition of adjuvant chemotherapy to endocrine therapy^11,12^, NET is emerging as an attractive option to optimize surgical outcomes without compromising survival in HR+HER2− breast cancer.

Axillary management after NET has not been well studied and the significance of residual axillary node disease after NET remains uncertain. In the upfront surgery setting, randomized trials have shown that sentinel lymph node biopsy (SLNB) alone is equivalent to axillary lymph node dissection (ALND) in terms of regional recurrence, disease-free and overall survival for patients with node-negative disease^13^, isolated tumor cells (ITCs)^14^, micrometastases^15,16^ and a low burden of macrometastatic disease^17,18^. Conversely, large retrospective series have shown that any residual disease on SLNB after NAC is associated with a high likelihood of additional nodal disease^19^. Further, both prospective and retrospective studies have shown that any amount of residual axillary disease after NAC, even ITCs (ypN0[i + ]) or micrometastases (ypN1mi), are associated with decreased disease-free survival (DFS) and overall survival (OS)^20,21^.

Given that patients undergoing NAC have received the majority of their systemic therapy prior to surgery, whereas those who receive NET have received only a short course of therapy (3–6 months)^2,22^ and continue to benefit from endocrine therapy in the adjuvant setting, we hypothesized that residual axillary node disease after NET would not carry the same prognostic implications as residual nodal disease after NAC. Here we use the National Cancer Data Base (NCDB) to explore the prognostic significance of residual nodal disease in both cN0 and cN1 patients selected for NET.

## Results

### Cohort characteristics

From 2010–2016, 4495 patients received NET for cT1–3N0–1M0 breast cancer. Median length of NET was 118 days, approximately 4 months (range 30–365 days). Median age was 65 years (range 23–90 years) and 966 (21.5%) patients had lobular tumor histology. The majority of patients had grade 1–2 tumors (3,753, 83.5%) and were clinically node negative (3,722, 82.8%). Adjuvant chemotherapy was used in 935 (20.8%) patients and 2,780 (61.8%) received adjuvant radiation therapy: 2,064 (82.3%) after breast conservation and 716 (25.8%) after mastectomy. Additional cohort characteristics are described in Table 1. Predictors of adjuvant therapy receipt are shown in Supplementary Table 1.

**Table 1:** Clinical characteristics for cT1–3N0–1 HR+HER2− patients selected for NET (*n* = 4495).

	Median (range)	Mean
Characteristic	*n* = 4495	%
Age, years	65 (23–90)	64.8 ± 11.9
Length of NET, days	118 (30–365)	126.8 ±76.5
Follow-up, months	36.2 (1.5–95.2)	39.9 ± 21.7
Nodes examined	3 (1–55)	6.2 ± 6.8
Nodes positive	0 (0–52)	1.54 ±3.6
Age	*N* = 4495	%
<50	524	11.7
50–69	2465	54.8
≥70	1506	33.5
Race		
Caucasian	3571	79.4
African American	406	9.0
Hispanic	344	7.7
API	146	3.2
Other	28	0.6
Histology		
Ductal	2948	65.6
Lobular	966	21.5
Mixed	581	12.9
Grade		
1	1325	29.5
2	2428	54.0
3	517	11.5
Unk	225	5.0
LVI		
No	3142	69.9
Yes	657	14.6
Unknown	696	15.5
Clinical T Stage		
cT1	1559	34.7
cT2	2285	50.8
cT3	651	14.5
Clinical N Stage		
cN0	3722	82.8
cN1	773	17.2
Surgery Type		
Lumpectomy	2509	55.8
Mastectomy	1986	44.2
Lymph Node Surgery		
SNB	2226	49.5
ALND	1445	32.1
Unknown	824	18.4
Pathologic Tumor Size		
ypT0	65	1.4
ypT1	2140	47.6
ypT2	1807	40.2
ypT3	465	10.3
ypTx	18	0.4
Pathologic N Stage		
ypN0	2510	55.8
ypN0 i+	99	2.2
ypN1mi	257	5.7
1–2 positive nodes	948	21.1
≥3 positive nodes	681	15.2
Adjuvant Treatment		
Radiation	2780	61.8
Chemotherapy	935	20.8

*ALND* axillary lymph node dissection, *API* Asian or Pacific Islander, *LVI* lymphovascular invasion, *NET* neoadjuvant endocrine therapy, *SNB* sentinel lymph node biopsy, *UNK* unknown.

### Residual nodal disease

After NET, 65 (1.4%) patients had pCR in the breast (54 [1.5%] cN0 and 11 [1.4%] cN1) and 54 (1.2%) patients had pCR in the breast and axillary nodes (51 [1.5%] cN0; 3 [0.4%] cN1).

Of 3,722 cN0 patients, 2,436 (65.4%) were ypN0, 325 (8.7%) had minimal residual nodal disease burden (92 [2.5%] ypN0[i + ]; 233 [6.3%] ypN1mi), 658 (17.7%) had low residual nodal disease burden (1–2 positive nodes), and 303 (8.1%) had high residual nodal burden (≥3 positive nodes) after NET. Of 773 cN1 patients, 74 (9.6%) were ypN0, 31 (4.0%) had minimal residual nodal disease burden (7 [0.9%] ypN0[i + ]; 24 [3.1%] ypN1mi), 290 (37.5%) had low residual nodal disease burden, and 378 (48.9%) had high residual nodal disease burden after NET, *p* < 0.01 (Table 2). There was no significant association between residual nodal disease burden and duration of NET in either cN0 or cN1 patients (Supplementary Table 2).

**Table 2:** Residual nodal disease burden after NET in HR+HER2− patients, stratified by clinical nodal status (*n* = 4495).

	cN0 (*n* = 3722)	cN1 (*n* = 773)
ypN0	2436 (65.4%)	74 (9.6%)
ypN0 [i + ]	92 (2.5%)	7 (0.9%)
ypN1mi	233 (6.3%)	24 (3.1%)
1–2 positive nodes	658 (17.7%)	290 (37.5%)
≥3 positive nodes	303 (8.1%)	378 (48.9%)
*P*-value		<0.01

*NET* neoadjuvant endocrine therapy.

### Overall survival by residual nodal disease burden

To test the hypothesis that minimal residual nodal disease (ypN0[i+] and ypN1mi) would not impact survival in NET patients, both unadjusted and adjusted OS analyses stratified by residual nodal disease burden were performed. Unadjusted Kaplan-Meier and adjusted Cox proportional hazards 5-year OS estimates are detailed in Table 3. There were no significant differences in OS between patients with ypN0, ypN0(i+), and ypN1mi disease after NET.

**Table 3:** Unadjusted Kaplan–Meier and adjusted Cox proportional hazards regression estimated 5-year OS, stratified by nodal burden (*n* = 3406).

	Unadjusted		Adjusted	
	5-year OS	*P*-value	5-year OS	*P*-value
ypN0	91.3%	Ref	93.8%	Ref
ypN0[i+]	95.7%	0.697	93.9%	0.959
ypN1mi	88.4%	0.413	92.4%	0.491
1–2 positive nodes	85.7%	0.001	88.9%	<0.001
≥3 positive nodes	75.1%	<0.001	80.7%	<0.001

*OS* overall survival.

**Fig. 1:**
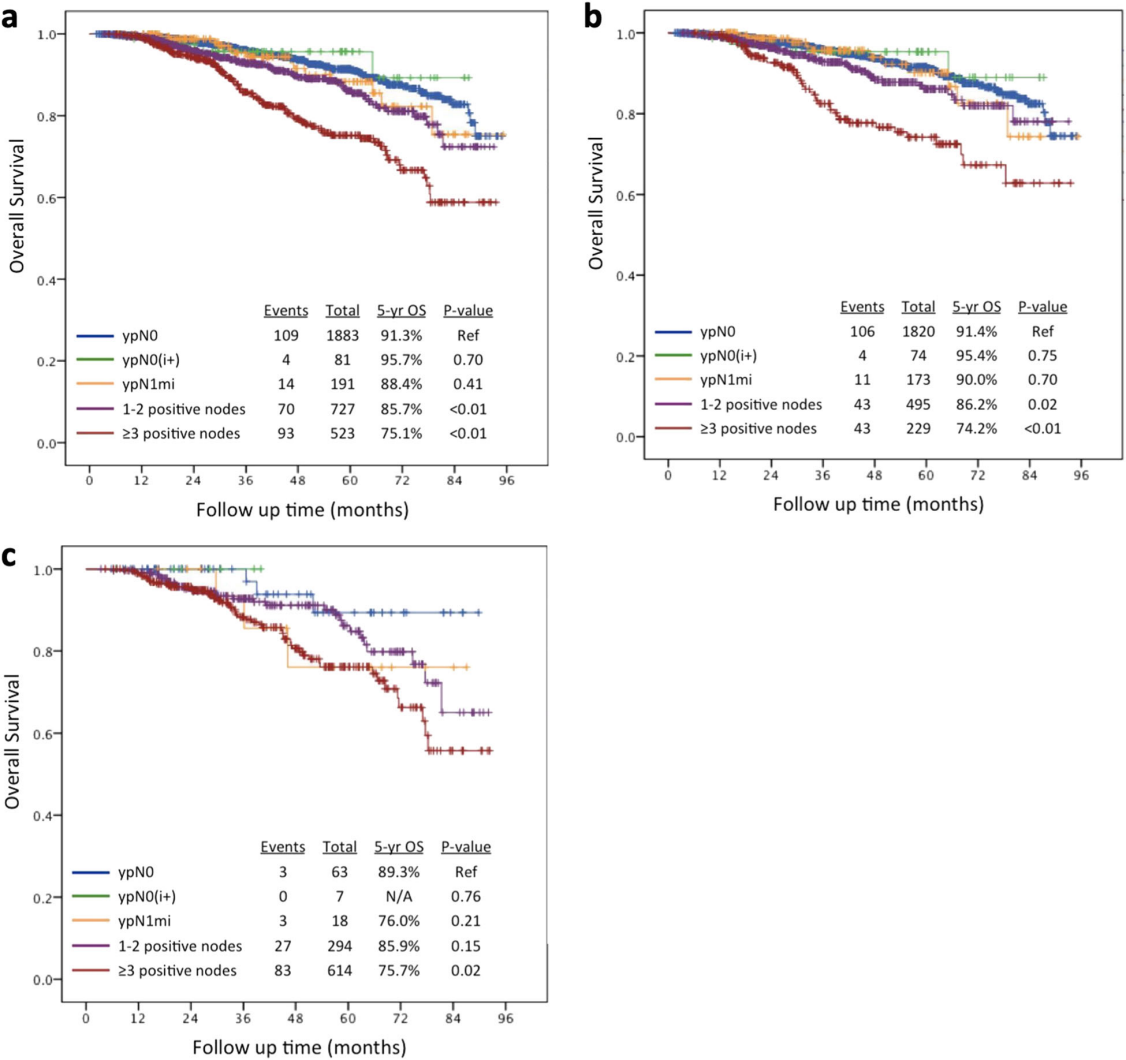
Kaplan–Meier overall survival curves by residual nodal disease burden for cT1–3N0–1 patients who received NET and surgery 2010–2015. **a** Entire cohort (*n* = 3406); **b** cN0 (*n* = 2782); **c** cN1 (*n* = 614).

Figure 1 demonstrates the unadjusted Kaplan–Meier curves for all NET patients (Fig. 1a), and stratified by clinical nodal status (Fig. 1b, c). In all groups, there were no statistically significant survival differences in patients with ypN0, or residual ypN0[i+] or ypN1mi, nodal disease.

Adjusted Cox proportional hazards analysis found that age ≥70 years (hazard ratio [HR] 4.60), increasing grade (HR 2.47 for grade 3), progesterone receptor (PR) negativity (HR 1.44), cT3 disease (HR 1.75), and residual macrometastatic nodal disease (HR 1.82 for 1–2 positive nodes; HR 3.37 for ≥3 positive nodes) were associated with increased mortality. Adjuvant therapies were associated with decreased mortality (HR 0.66 for chemotherapy; HR 0.69 for radiation). Minimal residual nodal disease burden (HR 0.97, 95% confidence interval [CI] 0.36–2.66 for ypN0[i+]; HR 1.22, 95% CI 0.69–2.15 for ypN1mi) after NET were not associated with decreased OS (Table 4).

**Table 4:** Cox proportional hazards analysis predicting mortality among HR+HER2− patients selected for NET (*n* = 3406).

Variable	Hazard ratio (95% CI)^a^	*P*-value
Age		
<50	1	Ref
50–69	1.73 (0.94–3.20)	0.078
≥70	4.60 (2.51–8.43)	<0.001
Race		
Caucasian	1	Ref
African American	1.19 (0.83–1.70)	0.356
Hispanic	0.56 (0.31–1.00)	0.050
Asian	0.45 (0.17–1.22)	0.117
Histology		
Ductal	1	Ref
Lobular	0.62 (0.45–0.86)	0.004
Mixed	0.62 (0.42–0.93)	0.019
Grade		
1	1	Ref
2	1.59 (1.17–2.18)	0.003
3	2.47 (1.67–3.66)	<0.001
PR		
Positive	1	Ref
Negative	1.44 (1.04–1.99)	0.029
Clin T		
cT1	1	Ref
cT2	1.16 (0.85–1.57)	0.361
cT3	1.75 (1.20–2.57)	0.004
Clin N		
cN0	1	Ref
cN1	0.75 (0.56–1.02)	0.063
Breast pCR		
No	1	Ref
Yes	0.93 (0.23–3.78)	0.920
LVI		
No	1	Ref
Yes	1.12 (0.84–1.51)	0.438
Node pathology		
ypN0	1	Ref
ypN0[i+]	0.97 (0.36–2.66)	0.959
ypN1mi	1.22 (0.69–2.15)	0.491
1–2 positive nodes	1.82 (1.31–2.53)	<0.001
≥3 positive nodes	3.37 (2.37–4.78)	<0.001
Chemotherapy		
Yes (vs. No)	0.66 (0.47–0.94)	0.021
Radiation		
Yes (vs. No)	0.69 (0.53–0.76)	0.007

^a^Adjusted for age, race, histology, grade, PR status, clin T, clin N, surgery type, breast pCR, LVI, nodal pathology, chemotherapy, radiation.

*CI* confidence interval, *HER2* human epidermal growth factor receptor 2, *HR* hormone receptor, *LVI* lymphovascular invasion, *NET* neoadjuvant endocrine therapy, *pCR* pathologic complete response, *PR* progesterone receptor.

To further test the hypothesis that minimal residual nodal burden in NET patients carries the same prognostic implications as in upfront surgery patients, we performed a propensity score analysis matching for clinical characteristics of age, race, clinical tumor and nodal stage, histology, grade, type of surgery, and presence of lymphovascular invasion (LVI). There were no differences in adjusted 5-year OS between NET and upfront surgery patients in any nodal burden category (Table 5).

**Table 5:** Overall survival outcomes by pathologic nodal group in ER+HER2− patients that were selected for NET and propensity score matched cohort of upfront surgery patients (*n* = 6812).

	NET (*n* = 3406)		Upfront surgery (*n* = 3406)		
	N events/Total	5-year OS^a^	N events/Total	5-year OS^a^	*P*-value
ypN0	109/1883	92.1%	92/1709	92.5%	0.621
ypN1[i+]	4/81	94.1%	10/107	87.3%	0.179
ypN1mi	14/191	89.3%	13/196	90.9%	0.661
1–2 positive nodes	70/727	88.1%	79/824	86.3%	0.319
≥3 positive nodes	93/523	78.6%	76/570	78.2%	0.975

^a^Adjusted for adjuvant chemotherapy and radiation.

*ER* estrogen receptor, *HER2* human epidermal growth factor receptor 2, *NET* neoadjuvant endocrine therapy, *OS* overall survival.

## Discussion

Although prospective randomized trials have demonstrated that NET and NAC result in similar clinical response rates and similar rates of breast conserving surgery in HR+HER2− disease^7^, NET use in the United States has largely been limited to select populations and management of the axilla after NET has not been well studied. As such, surgeons generally extrapolate guidelines for management of the axilla after NET from the NAC literature. In this cohort of cN0 and cN1 HR+HER2− patients treated with NET, the distribution of residual nodal disease varied significantly by clinical node status with the majority of cN0 patients having negative nodes or minimal residual nodal disease and nearly 50% of cN1 patients having 3 or more positive nodes. Overall, 8% of cN0 and 4% of cN1 patients had either ITCs (2.5% and 0.9%, respectively) or micrometastases (6.3% and 3.1%) on final pathology and this minimal residual nodal disease was not associated with inferior OS when compared to those with node-negative disease, suggesting an opportunity to de-escalate axillary treatment after NET.

Similar to our findings after NET, clinical trials in the upfront surgery setting have demonstrated that survival outcomes are similar in patients with negative nodes or minimal nodal disease^14,20,23^. The American College of Surgeons Oncology Group Z0010 trial followed 5,210 patients with cT1–2N0 breast cancer treated with BCT and SLNB. Central immunohistochemical staining found ITCs in 10.5% of SLN that were negative by hematoxylin-eosin staining. At five years, there were no significant differences in DFS or OS in patients with ITCs compared to those with negative nodes despite the fact that both patients and physicians were blinded to this outcome^14^. An analysis of occult ITCs in the National Surgical Adjuvant Breast and Bowel Project (NSABP) B-32 trial, which randomized 5,611 patients with pathologically node negative breast cancer to SLNB or ALND, reported that occult ITCs were present in 15.9% of patients and were associated with a small statistical, but not clinically significant, decrease in OS at 5 years (94.6% vs. 95.8%)^23^. In addition, more modern trials of SLNB alone vs ALND in patients with micrometastatic disease found on SLNB in the upfront surgery setting, have demonstrated no difference in long-term outcomes based on the performance of ALND. The International Breast Cancer Study Group 23–01 trial randomized 934 patients with cT1–2N0 breast cancer undergoing BCT or mastectomy and found to have micrometastatic disease on SLNB to either ALND or observation. While 13% of patients in the ALND arm had additional involved axillary nodes, at 10 years of follow up, there were no differences in DFS between the two arms^15^. Similarly, the Spanish AATRM trial randomized patients with tumors <3.5 cm and micrometastatic disease on SLNB to ALND or observation; 13% of patients in the ALND arm had additional axillary disease identified, yet there were no differences in DFS at 5 years of follow up^16^.

In contrast, even minimal residual nodal disease after NAC may in fact impact survival. In previous work from our group we examined the prognostic significance of residual nodal disease after NAC in 967 patients with cT1–4N0–1 disease treated from 2002–2014 at our institution, and a similar cohort of 35,536 patients treated with NAC as reported to the NCDB. In both cohorts survival outcomes were significantly decreased in patients with any residual nodal disease burden, including ITCs and micrometastases, when compared to patients with node-negative disease^19^. Exploratory analysis of the landmark NSABP B-18 trial, which randomized 1,523 patients to preoperative or postoperative chemotherapy, also demonstrated that micrometastatic nodal disease was associated with inferior DFS and OS in patients treated with preoperative chemotherapy; whereas this association was not seen in upfront surgery patients^20^.

The lack of a survival detriment seen in patients with ITCs and micrometastases after NET suggests that outcomes of patients after NET more closely mirror those of upfront surgery patients rather than those having NAC. The propensity score analysis with upfront surgery patients, demonstrating no differences in survival for any category of residual nodal disease suggests that clinical management strategies that mirror those used in the upfront surgery setting (such as omitting ALND in patients with 1–2 positive nodes) may be more appropriate following NET rather than strategies used after NAC. Our findings that approximately 30% of patients presenting with cN0 disease had node-positive disease after NET are consistent with reported rates of axillary metastases in upfront surgery patients^13,17,18^, including those specifically with HR+HER2− disease^24^. Further, the observation that only 8% of cN0 patients treated with NET had 3 or more positive nodes lends support to using SLNB to identify patients with a low residual nodal disease burden and an opportunity to de-escalate axillary surgery after NET.

Acknowledging that this NCDB study cohort largely represents a group of patients with early stage, low grade, clinically node negative disease; predictors of mortality were consistent with well known factors, including increasing grade, T category, and increasing number of pathologically positive nodes^25^. Although the selection bias for NET in this cohort may limit the generalizability of our findings, in the era of genomic assays for treatment selection there is likely a role for increased utilization of NET to achieve breast conservation in the broader population that will not benefit from chemotherapy, thus providing an opportunity to further refine axillary management strategies in this patient population.

There are several limitations of this study. The NCDB does not record recurrence or DFS, and thus OS is the only available outcome measure. The follow up time is relatively short and longer follow up is needed as HR+HER2− patients may recur as late as 10 years after initial treatment^26^. Details on the type of endocrine therapy as well as the duration of endocrine therapy after surgery are not known. Detailed information on the intent of axillary surgery (SLNB vs. ALND) was not available until 2012, and thus was not able to be specifically accounted for in this analysis. There are also a relatively small number of patients in the cN1 subgroup, therefore, the analysis may be underpowered to predict OS in this group. Further, it is further unknown if cN1 patients had clinically palpable or biopsy-proven nodal disease making the results more difficult to interpret in this group.

In conclusion, these data suggest that, in patients selected for NET, minimal residual nodal disease burden (ITCs or micrometastases) have no impact on OS. Further, survival outcomes for NET patients are more similar to patients undergoing upfront surgery than to patients receiving NAC. While further study is needed, the adoption of axillary management strategies utilized in upfront surgery patients may be more appropriate in patients receiving NET.

## Methods

### Data source

The NCDB is a joint project of the American Cancer Society and the American College of Surgeons Commission on Cancer, which captures approximately 70% of new cancer diagnoses in the United States. Variables include patient demographics (age, race, comorbidities), tumor characteristics (histology, grade, lymphovascular invasion, clinical and pathologic stage), treatment characteristics (surgery, radiation, systemic therapy, and timing of therapies), and overall survival. Breast cancer site-specific factors include estrogen receptor, progesterone receptor and HER2 receptor status. Data are compliant with the Health Insurance Portability and Accountability Act (HIPAA). As all data are de-identified, this study was deemed to be IRB exempt by the Brigham and Women’s Hospital Institutional Review Board.

### Cohort selection

Selection criteria included female patients with minimal comorbidities (Charlson-Deyo index of <2) who underwent NET for HR+HER2− clinical stage T1–3N0–1M0 breast cancer from 2010–2016 followed by breast and axillary surgery. Patients with missing information regarding systemic therapy or nodal pathology were excluded. Patients treated with concurrent or subsequent neoadjuvant chemotherapy were also excluded (Supplementary Table 3). NET was defined as endocrine therapy duration for at least 30 days and no longer than 1 year prior to the date of surgery. American Joint Committee on Cancer 7^th^ edition staging was used for determination of clinical and pathologic stage groups^27^.

Clinical N1 disease is defined in the NCDB as the presence of features highly suspicious for malignancy in movable ipsilateral level I-II axillary lymph nodes on imaging or physical exam, or the presence of biopsy-proven metastases. The methods of detection of cN1 disease are not specified. LVI was considered present if identified in any pathology report (biopsy or final pathology). All patients had lymph nodes examined for pathology. Residual nodal disease burden was defined as the number of positive lymph nodes. ITCs and micrometastases were defined as recorded in the pathologic nodal stage variable in the NCDB. Five categories of residual nodal burden were examined after NET: ypN0, ypN0[i+], ypN1mi, 1–2 positive nodes, and ≥3 positive nodes). Minimal nodal disease burden was defined as ypN0[i+] or ypN1mi. Low residual nodal disease burden was defined as 1–2 macroscopically positive nodes, and high residual nodal disease burden was defined as ≥3 macroscopically positive nodes.

### Statistical analysis

The primary endpoint was OS after NET stratified by the residual axillary nodal disease burden. Secondary endpoints included the distribution of residual nodal disease burden by clinical nodal status; predictors of adjuvant treatment; and predictors of mortality among the patient cohort. Descriptive statistics were used to examine the residual nodal disease burden. Multivariable logistic regression was used to identify independent predictors of adjuvant treatment (radiation and chemotherapy) after NET. Kaplan-Meier and Cox proportional hazards analysis were used to estimate 5-year OS stratified by residual nodal disease burden for patients diagnosed in 2010–2015. Kaplan-Meier curves were generated for each residual nodal disease burden category compared to ypN0. Log-rank tests were used to calculate p-values. Hazard Ratios (HR) > 1 on Cox regression were considered associated with higher risk of mortality. A matched cohort of upfront surgery patients was identified to compare OS using propensity score matching on the clinical characteristics of age, race, clinical tumor and nodal stage, histology, grade, type of surgery, and presence of LVI. The matched survival analysis was adjusted for adjuvant treatment (chemotherapy and radiation). Survival for 2016 was not available due to lack of follow-up time. A *p*-value < 0.05 was considered statistically significant; all *p*-values were 2-sided. All analyses were performed using SPSS statistical software version 23.0 (IBM Corp., Armonk, NY).

### Reporting summary

Further information on research design is available in the Nature Research Reporting Summary linked to this article.

## Supplementary information

Supplementary Information

Reporting Summary

## Data Availability

The data generated and analyzed during this study are described in the following data record: 10.6084/m9.figshare.12651773^28^. The data from the National Cancer Data Base, analyzed during the current study, are not publicly available. The data will be made available to researchers at the Commission on Cancer (CoC) centres, who have completed an application form and a Data Usage Agreement. Please contact NCDB_PUF@facs.org for data access requests.
