# Supplementary material for: Prognostic significance of residual nodal disease after neoadjuvant endocrine therapy for hormone receptor-positive breast cancer
Source: NPJ Breast Cancer. 2020 Aug 13;6:35. doi: 10.1038/s41523-020-00177-6 (PMC7426953; doi:10.1038/s41523-020-00177-6)
Supplement: Supplementary file 1 — Supplementary Information [file 41523_2020_177_MOESM1_ESM.pdf]

Prognostic Significance of Residual Nodal Disease after Neoadjuvant Endocrine Therapy for Hormone Receptor-Positive Breast Cancer

Olga Kantor, MD, MS; Stephanie Wong, MD; Anna Weiss, MD; Otto Metzger, MD; Elizabeth A Mittendorf, MD, PhD; Tari A King, MD

Supplementary Tables: 3

Supplementary Table 1. Multivariable analysis of predictors of receiving chemotherapy and radiotherapy in HR+HER2- patients selected for NET (n=4495).

	Predictors of Chemotherapy		Predictors of Radiation	
**Variable**	**OR (95% CI)***	**P-value**	**OR (95% CI)***	**P-value**
Age				
<50	12.90 (9.45-17.60)	<0.01	2.39 (1.84-3.09)	<0.01
50-69	6.64 (5.23-8.43)	<0.01	2.58 (2.18-3.04)	<0.01
≥70	1	Ref	1	Ref
Race				
Caucasian	1	Ref	1	Ref
African American	0.88 (0.65-1.18)	0.39	1.07 (0.82-1.40)	0.60
Hispanic	1.34 (0.99-1.81)	0.06	1.02 (0.77-1.36)	0.88
Asian	0.71 (0.43-1.19)	0.19	0.90 (0.59-1.36)	0.62
Histology				
Ductal	1	Ref	1	Ref
Lobular	1.04 (0.83-1.31)	0.71	1.37 (1.12-1.67)	<0.01
Mixed	1.07 (0.83-1.38)	0.62	0.95 (0.76-1.20)	0.67
Grade				
1	1	Ref	1	Ref
2	1.47 (1.19-1.81)	<0.01	0.99 (0.93-1.18)	0.91
3	3.09 (2.31-4.13)	<0.01	0.81 (0.62-1.06)	0.13
HR Status				
ER+PR+	1	Ref	1	Ref
ER+PR-	2.18 (1.66-2.85)	<0.01	0.94 (0.74-1.21)	0.63
ER-PR+	1.43 (0.10-21.20)	0.80	4.69 (0.25-89.71)	0.31
Clin T				
cT1	1	Ref	1	Ref
cT2	0.91 (0.73-1.14)	0.42	1.23 (1.02-1.48)	0.03
cT3	0.82 (0.60-1.13)	0.22	2.21 (1.66-2.95)	<0.01
Clin N				
cN0	1	Ref	1	Ref
cN1	0.97 (0.77-1.22)	0.80	0.97 (0.77-1.23)	0.43
LVI				
No	1	Ref	1	Ref
Yes	1.22 (0.97-1.53)	0.09	0.94 (0.74-1.18)	0.59
Breast Surgery				
BCS	1	Ref	1	Ref
Mastectomy	1.13 (0.93-1.36)	0.20	0.05 (0.04-0.06)	<0.01
Path T				
pT0 (breast pCR)	0.26 (0.08-0.92)	0.04	0.71 (0.38-1.32)	0.27
pT1	1	Ref	1	Ref
pT2	1.48 (1.20-1.84)	<0.01	1.08 (0.89-1.32)	0.42
pT3	2.07 (1.48-2.91)	<0.01	2.04 (1.48-2.82)	<0.01
pT4	0.29 (0.09-0.94)	0.04	1.50 (0.70-3.19)	0.30
Node Pathology				

ypN0	1	Ref	1	Ref
ypN0[i+]	2.29 (1.34-3.91)	<0.01	1.34 (0.80-2.23)	0.26
ypN1mi	2.73 (1.91-3.91)	<0.01	1.76 (1.25-2.48)	<0.01
1-2 positive nodes	5.52 (4.39-6.94)	<0.01	2.71 (2.17-2.28)	<0.01
≥3 positive nodes	13.27 (10.02-17.58)	<0.01	5.98 (4.53-7.89)	<0.01

Abbreviations: BCS, breast-conserving surgery; CI, confidence interval; ER, estrogen receptor; HER2, human epidermal growth factor receptor 2; HR, hormone receptor; LVI, lymphovascular invasion; NET, neoadjuvant endocrine therapy; OR, odds ratio; pCR, pathologic complete response; PR, progesterone receptor

Supplementary Table 2. Residual nodal burden in HR+HER2- patients selected for NET, stratified by clinical nodal status and duration of NET (n=4495).

	cN0 (n=3722)		cN1 (n=773)	
	NET <=180 days (n=2952)	NET >180 days (n=770)	NET <=180 days (n=560)	NET >180 days (n=213)
ypN0	1961 (66.1%)	485 (63.0%)	50 (8.9%)	24 (11.3%)
ypN0[i+]	76 (2.6%)	16 (2.1%)	4 (0.7%)	3 (1.4%)
ypN1mi	184 (6.2%)	49 (6.4%)	15 (2.7%)	9 (4.2%)
1-2 positive nodes	512 (17.3%)	146 (19.0%)	222 (39.6%)	68 (31.9%)
≥3 positive nodes	229 (7.8%)	74 (20.7%)	269 (48.0%)	109 (51.2%)
P-value	0.29		0.23	

Abbreviations: HER2, human epidermal growth factor receptor 2; HR, hormone receptor; NET, neoadjuvant endocrine therapy

Supplementary Table 3. NCDB Selection Criteria

**Selection Criteria**	**N**
All patients 2004-2016	2696734
Female	2672744
Treated at reporting facility	2563887
No previous cancer, unilateral	1925355
Breast cancer histology, exclude inflammatory	1853274
cT1-3N0-1	979328
Had breast and LN surgery, known path stage	899517
HR+	739932
Her2-	470663
Minimal comorbidities (Charlson-Deyo Index <2)	455182
Neoadjuvant systemic therapy	23303
Neoadjuvant endocrine therapy (at least 30 days, no longer than 1 year) without neoadjuvant chemotherapy	**4495**

Abbreviations: HER2, human epidermal growth factor receptor 2; HR, hormone receptor; LN, lymph node; NCDB, National Cancer Data Base
