# Supplementary material for: Prognostic significance of residual nodal disease after neoadjuvant endocrine therapy for hormone receptor-positive breast cancer
Source: NPJ Breast Cancer. 2020 Aug 13;6:35. doi: 10.1038/s41523-020-00177-6 (PMC7426953; doi:10.1038/s41523-020-00177-6)
Supplement: Supplementary file 2 — Reporting Summary [file 41523_2020_177_MOESM2_ESM.pdf]

nature research

Corresponding author(s): Tari King

Last updated by author(s): May 1, 2020

Reporting Summary

Nature Research wishes to improve the reproducibility of the work that we publish. This form provides structure for consistency and transparency in reporting. For further information on Nature Research policies, see Authors & Referees and the Editorial Policy Checklist.

Statistics

For all statistical analyses, confirm that the following items are present in the figure legend, table legend, main text, or Methods section.

n/a	Confirmed	
☐	☒	The exact sample size (n) for each experimental group/condition, given as a discrete number and unit of measurement
☒	☐	A statement on whether measurements were taken from distinct samples or whether the same sample was measured repeatedly
☐	☒	The statistical test(s) used AND whether they are one- or two-sided<br>*Only common tests should be described solely by name; describe more complex techniques in the Methods section.*
☐	☒	A description of all covariates tested
☐	☒	A description of any assumptions or corrections, such as tests of normality and adjustment for multiple comparisons
☐	☒	A full description of the statistical parameters including central tendency (e.g. means) or other basic estimates (e.g. regression coefficient) AND variation (e.g. standard deviation) or associated estimates of uncertainty (e.g. confidence intervals)
☐	☒	For null hypothesis testing, the test statistic (e.g. F, t, r) with confidence intervals, effect sizes, degrees of freedom and P value noted<br>*Give P values as exact values whenever suitable.*
☒	☐	For Bayesian analysis, information on the choice of priors and Markov chain Monte Carlo settings
☒	☐	For hierarchical and complex designs, identification of the appropriate level for tests and full reporting of outcomes
☒	☐	Estimates of effect sizes (e.g. Cohen's d, Pearson's r), indicating how they were calculated

Our web collection on statistics for biologists contains articles on many of the points above.

Software and code

Policy information about availability of computer code

Data collection	No software was used for data collection.
Data analysis	SPSS version 23 was used for analysis.

For manuscripts utilizing custom algorithms or software that are central to the research but not yet described in published literature, software must be made available to editors/reviewers. We strongly encourage code deposition in a community repository (e.g. GitHub). See the Nature Research guidelines for submitting code & software for further information.

Data

Policy information about availability of data

All manuscripts must include a data availability statement. This statement should provide the following information, where applicable:

- Accession codes, unique identifiers, or web links for publicly available datasets
- A list of figures that have associated raw data
- A description of any restrictions on data availability

Data from the NCDB is publicly available with permission.

Field-specific reporting

Please select the one below that is the best fit for your research. If you are not sure, read the appropriate sections before making your selection.

☐ Life sciences ☒ Behavioural & social sciences ☐ Ecological, evolutionary & environmental sciences

For a reference copy of the document with all sections, see nature.com/documents/nr-reporting-summary-flat.pdf

Behavioural & social sciences study design

All studies must disclose on these points even when the disclosure is negative.

Study description	Quantitative retrospective cohort design.
Research sample	Breast cancer patients from the National Cancer DataBase, which encompasses 70% of newly diagnosed breast cancer cases at Commission on Cancer accredited centers.
Sampling strategy	This was a retrospective cohort study; we selected a specific group of patients for analysis.
Data collection	Data collection was through the National Cancer DataBase and not by the individual investigators.
Timing	Collection dates were 1/1/2010-12/31/2016 based on the dates of diagnosis included in the 2016 National Cancer DataBase breast file.
Data exclusions	Study inclusion criteria is detailed in the methods; we included only female patients with clinical stage T1-3N0-1M0 hormone receptor-positive, HER2-negative breast cancer who underwent neoadjuvant endocrine therapy followed by breast and axillary surgery. Patients with unknown pathologic data and those with neoadjuvant endocrine therapy course outside of 30-365 days were excluded.
Non-participation	N/A - not human subject research
Randomization	N/A - not human subject research

Reporting for specific materials, systems and methods

We require information from authors about some types of materials, experimental systems and methods used in many studies. Here, indicate whether each material, system or method listed is relevant to your study. If you are not sure if a list item applies to your research, read the appropriate section before selecting a response.

Materials & experimental systems

n/a	Involved in the study	
☒	☐	Antibodies
☒	☐	Eukaryotic cell lines
☒	☐	Palaeontology
☒	☐	Animals and other organisms
☒	☐	Human research participants
☐	☒	Clinical data

Methods

n/a	Involved in the study	
☒	☐	ChIP-seq
☒	☐	Flow cytometry
☒	☐	MRI-based neuroimaging

Clinical data

Policy information about clinical studies

All manuscripts should comply with the ICMJE guidelines for publication of clinical research and a completed CONSORT checklist must be included with all submissions.

Clinical trial registration	N/A
Study protocol	Described in detail in the methods and supplemental material.
Data collection	Data collection for the National Cancer DataBase is done locally at each participating hospital by cancer registrars.
Outcomes	Our primary outcome was overall survival; this was defined as time from date of diagnosis to date of death or last follow up.
